# Consumption of Fermented Foods Is Associated with Systematic Differences in the Gut Microbiome and Metabolome

**DOI:** 10.1128/mSystems.00901-19

**Published:** 2020-03-17

**Authors:** Bryn C. Taylor, Franck Lejzerowicz, Marion Poirel, Justin P. Shaffer, Lingjing Jiang, Alexander Aksenov, Nicole Litwin, Gregory Humphrey, Cameron Martino, Sandrine Miller-Montgomery, Pieter C. Dorrestein, Patrick Veiga, Se Jin Song, Daniel McDonald, Muriel Derrien, Rob Knight

**Affiliations:** a Biomedical Sciences Graduate Program, University of California San Diego, La Jolla, California, USA; b Department of Pediatrics, School of Medicine, University of California San Diego, La Jolla, California, USA; c Division of Biostatistics, University of California San Diego, La Jolla, California, USA; d Skaggs School of Pharmacy and Pharmaceutical Sciences, University of California San Diego, La Jolla, California, USA; e Bioinformatics and Systems Biology Program, University of California San Diego, La Jolla, California, USA; f IT&M Innovation on behalf of Danone Nutricia Research, Palaiseau, France; g Danone Nutricia Research, Palaiseau, France; h Department of Bioengineering, Jacobs School of Engineering, University of California San Diego, La Jolla, California, USA; i Center for Microbiome Innovation, University of California San Diego, La Jolla, California, USA; j Department of Computer Science and Engineering, University of California San Diego, La Jolla, California, USA; Princeton University

**Keywords:** microbiome, fermented food

## Abstract

Lifestyle factors, such as diet, strongly influence the structure, diversity, and composition of the microbiome. While we have witnessed over the last several years a resurgence of interest in fermented foods, no study has specifically explored the effects of their consumption on gut microbiota in large cohorts. To assess whether the consumption of fermented foods is associated with a systematic signal in the gut microbiome and metabolome, we used a multi-omic approach (16S rRNA amplicon sequencing, metagenomic sequencing, and untargeted mass spectrometry) to analyze stool samples from 6,811 individuals from the American Gut Project, including 115 individuals specifically recruited for their frequency of fermented food consumption for a targeted 4-week longitudinal study. We observed subtle but statistically significant differences between consumers and nonconsumers in beta diversity as well as differential taxa between the two groups. We found that the metabolome of fermented food consumers was enriched with conjugated linoleic acid (CLA), a putatively health-promoting molecule. Cross-omic analyses between metagenomic sequencing and mass spectrometry suggest that CLA may be driven by taxa associated with fermented food consumers. Collectively, we found modest yet persistent signatures associated with fermented food consumption that appear present in multiple -omic types which motivate further investigation of how different types of fermented food impact the gut microbiome and overall health.

**IMPORTANCE** Public interest in the effects of fermented food on the human gut microbiome is high, but limited studies have explored the association between fermented food consumption and the gut microbiome in large cohorts. Here, we used a combination of omics-based analyses to study the relationship between the microbiome and fermented food consumption in thousands of people using both cross-sectional and longitudinal data. We found that fermented food consumers have subtle differences in their gut microbiota structure, which is enriched in conjugated linoleic acid, thought to be beneficial. The results suggest that further studies of specific kinds of fermented food and their impacts on the microbiome and health will be useful.

## Abstract (precis)

Public interest in the effects of fermented food on the human gut microbiome is high, but limited studies have explored the association between fermented food consumption and the gut microbiome in large cohorts. Here, we used a combination of omics-based analyses to study the relationship between the microbiome and fermented food consumption in thousands of people using both cross-sectional and longitudinal data. We found that fermented food consumers have subtle differences in their gut microbiota structure, which is enriched in conjugated linoleic acid, thought to be beneficial. The results suggest that further studies of specific kinds of fermented food and their impacts on the microbiome and health will be useful.

## INTRODUCTION

Fermentation is an ancient process of food preparation dating from the introduction of agriculture and animal husbandry during the Neolithic period approximately 10,000 years ago. Advantages of food fermentation include improvements in food preservation, food safety, nutritional value, and organoleptic quality resulting from the activity of microbial ecosystems (bacteria and yeast) (1). Fermentation can be applied to a range of food types, including meat, fish, milk, vegetables, beans, cereals, and fruits, and occurs spontaneously from the original ingredients or environment or is controlled by the addition of specific starters such as lactic acid bacteria (LAB) (2). These bacteria are commonly detected in fermented food, mostly including *Lactobacillus*, *Streptococcus*, *Lactococcus*, and *Leuconostoc*, but other bacteria as well as yeast and fungi are also involved in food fermentations (3). In addition to microbial diversity, the number of microorganisms present in fermented foods varies between food type, process, and storage. A survey of diverse fermented food products suggested that the count of viable lactic acid bacteria usually reaches at least 10^6^ cells/ml (4). Recovery of viable bacterial and fungal species ingested through fermented food has been observed in subjects who consume an animal-based diet (5). Moreover, metabolites generated from fermentation, including lactic acid, vitamins, and exopolysaccharides, are thought to exert health benefits (6). A recent study reported that d-phenyllactic acid, produced by LAB, interacts with the human host through the activation of hydroxycarboxylic acid receptor 3 (HCA3) and is involved in the regulation of immune functions and energy homeostasis under changing metabolic and dietary conditions (7).

Due to their supposed health benefits (6), there has been a resurgence of interest in consumption of fermented foods in Western society. To date, many of the studies focused on the health benefits of fermented food intake have been mostly focused on yogurt, consumption of which is associated with better metabolic parameters in large American cohorts (8, 9). Similarly, high intake of fermented foods has been associated with a lower prevalence of atopic dermatitis in a Korean population (10), and another study found consumption of miso and natto to be inversely associated with high blood pressure in a Japanese population (11).

While we know that both short- and long-term dietary intake affects the structure, function, and activity of the human gut microbiome (5, 12–16), and a few studies have explored the response of gut microbiota to a single type of fermented food (recently reviewed in reference 17), no study has explored the functional capacity of the gut microbiota of fermented food consumers. Intervention studies, which are often underpowered for analysis of the gut microbiome response, are complemented by studies of population-based cohorts, which due to large sample sizes have the advantage of capturing large amounts of microbial variation and enable us to disentangle the contributions of host and environmental factors such as diet (18–21).

To address the hypothesis that fermented food consumption is associated with compositional or functional changes in the human gut microbiome, we analyzed a subset of the American Gut Project (AGP) cohort based on self-reported consumption of fermented foods, and in particular, fermented plants. We also explored the longitudinal stability and function of the gut microbiota using untargeted high-performance liquid chromatography–tandem mass spectrometry (HPLC-MS/MS) and 16S rRNA amplicon sequencing, as well as shotgun sequencing on a subset of subjects at a single time point.

## RESULTS

### Demographic and dietary assessments of fermented plant consumers and nonconsumers.

**FIG 1:**
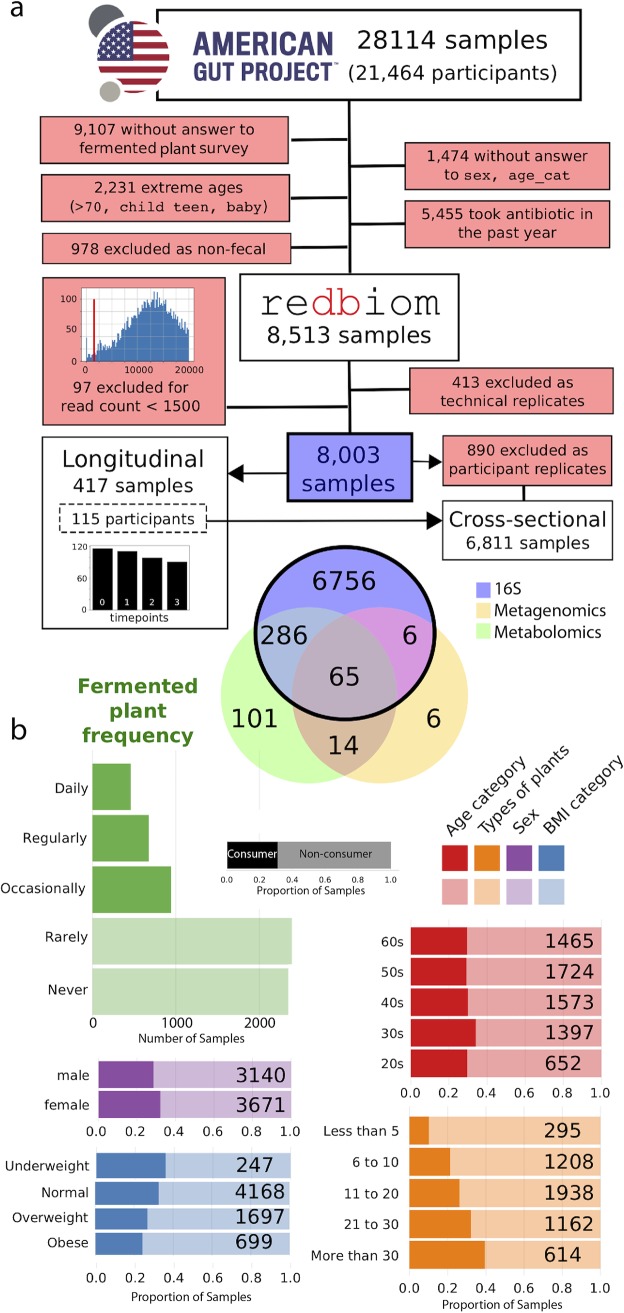
Cohort overview, sample filtering, and metadata exploration. (a) Data filtering process and the number of samples analyzed by 16S rRNA gene sequencing, metabolomics, and shotgun metagenomics and the resulting number of samples in the cross-sectional and longitudinal cohorts. (b) Distribution of some metadata categories (demographic and diet) in the cross-sectional cohort between consumers and nonconsumers. Darker colors denote consumers; lighter colors denote nonconsumers. The consumer and nonconsumer groups were defined by the “fermented plant frequency” questionnaire.

To explore the differences in the gut microbiome between fermented food consumers and nonconsumers, we analyzed 16S rRNA sequencing data from 28,114 samples from 21,464 individuals in the AGP (Fig. 1a). After filtering (see Materials and Methods), 6,811 participants were retained, and here are referred to as the cross-sectional cohort (Fig. 1a). One hundred fifteen of these participants were initially recruited for a concurrent longitudinal assessment which is discussed in detail below. Participants were identified as “consumers” or “nonconsumers” depending on the frequency of fermented plants that they reported consuming. The fermented plant frequency question is in the standard AGP questionnaire that every participant answered, and while the language may not have allowed for the capture of all fermented foods, this represented the most efficient way to delineate consumers and nonconsumers. We considered consumers to be those who reported eating fermented plants “daily,” “regularly (3 to 5 times/week),” or “occasionally (1 to 2 times/week)” and nonconsumers to be those who reported eating fermented plants either “rarely (less than once/week)” or “never” (Fig. 1b). A 30.5% proportion of participants were considered consumers, of which most (45.3%) were occasional consumers. Consumer and nonconsumer cohorts were composed of slightly differing demographic groups. For example, while consumers were significantly younger than nonconsumers, the difference was modest (47 versus 47.61 years, respectively), with a higher proportion of participants in their 30s (23.0% versus 19.4%; chi-square test = 11.08, *P* = 0.03) (Fig. 1b). Similarly, the consumer group was composed of a modestly higher proportion of females (56.8% versus 52.6%; chi-square test = 9.60, *P* = 0.002) and a higher proportion of participants with a normal body mass index (BMI) between 18.5 and 25 (65.6% versus 59.3%; chi-square test = 35.93, *P* ≪ 0.001), with an average BMI of 23.9 and 24.8, respectively. Consumers also reported eating a greater diversity of plants (>20) (29.7% versus 24.5%; chi-square test = 126.96, *P* ≪ 0.001). In addition, because alcohol may be an end product of a fermentation process and might be a confounding factor associated with gut microbiota variation, we verified that alcohol consumption was not associated with fermented plant consumption (81.7% versus 82.6%, chi-square test = 0.76, *P* value = 0.38).

Statistically significant differences in mean total carbohydrate and fat intake (grams/day and percentage of energy) and percentage of energy from protein, as estimated by the food frequency questionnaire (FFQ), were observed between fermented plant consumers and nonconsumers, while total energy (kilocalories/day), dietary fiber (grams/day), and protein (grams/day) intake did not differ (see Table S1 in the supplemental material). There was no significant difference in overall diet quality observed, as assessed by the Healthy Eating Index (HEI-2010; Mann-Whitney U = 223409, *P* value = 0.094; Fig. S1A), despite the differences in the consumption of fermented plants and number of plant types between consumers and nonconsumers; this nonsignificant difference in total HEI-2010 scores between consumers and nonconsumers (71.29 versus 71.53, respectively) suggests similar intake of dietary patterns relatively high in quality. It should be noted that the mean total HEI-2010 score for both consumers and nonconsumers is above the national average (58.27) for U.S. adults aged 18 to 64 years based on 2011–2012 National Health and Nutrition Examination (NHANES) data (22). This suggests that the cohort in our study has a diet pattern that better aligns to the Dietary Guidelines for Americans than that of average American adults. Additionally, it has been shown that higher HEI scores are associated with higher income and education levels (23, 24), thereby suggesting that the higher total HEI scores observed in this AGP cohort may reflect higher-than-average socioeconomic status and education level as previously observed (25).

10.1128/mSystems.00901-19.1 FIG S1 (A) Healthy Eating Index-2010 scores of consumers (*n* = 483) and nonconsumers (*n* = 966). (B) Taxonomic differences (16S) between consumers and nonconsumers in the cross-sectional cohort. Unweighted UniFrac principal-coordinate analysis (PCoA) colored by consumers (red) and nonconsumers (teal). Beta diversity (PERMANOVA), unweighted UniFrac, pseudo-*F* test statistic = 3.677, *P* = 0.001; weighted UniFrac, pseudo-*F* test statistic = 2.163, *P* = 0.058. Alpha diversity (Kruskal-Wallis), Faith’s PD, *H* = 4,908,194, *P* value = 0.9565; Shannon, *H* = 4947661, *P* value = 0.6356; richness, *H* = 4831862, *P* value = 0.793. (C) Differentially abundant taxa associated with consumers (“set 1 microbes,” Table S3) and nonconsumers (“set 2 microbes,” Table S3) identified by Songbird. The log ratio of set 1 to set 2 is significantly different (*t* test *P* = 0.00065, *t* = 3.6367, *df* = 50.76, Cohen’s *D* = 0.9998). Download FIG S1, PDF file, 1.9 MB. Copyright © 2020 Taylor et al. 2020 Taylor et al. This content is distributed under the terms of the Creative Commons Attribution 4.0 International license.

10.1128/mSystems.00901-19.7 TABLE S1 Estimated daily nutrient intake for fermented plant consumers and nonconsumers. Data are presented as group mean ± SD; (min, max). *P* values were obtained via Mann-Whitney U test. Abbreviations: d, day; NS, nonsignificant. Download Table S1, PDF file, 0.1 MB. Copyright © 2020 Taylor et al. 2020 Taylor et al. This content is distributed under the terms of the Creative Commons Attribution 4.0 International license.

### Gut microbiome composition in fermented plant consumers and nonconsumers.

Examining unweighted UniFrac distances (26), we observed a statistically significant difference in the overall gut microbial communities between consumers and nonconsumers (Fig. S1B, permutational multivariate analysis of variance [PERMANOVA] pseudo-*F*-statistic = 3.677, *P* = 0.001). The comparison of nonconsumers with occasional consumers results in a weaker group separation (*F*-statistic = 2.233, *P* value = 0.001) than with regular or daily consumers (*F*-statistics = 3.512 and 3.246, respectively; *P* values = 0.001), suggesting a dose dependence for the frequency of fermented plant consumption on the gut microbiome. However, there was no dose dependence with frequency of types of plants between consumers and nonconsumers (unweighted UniFrac distances between consumers and nonconsumers versus the frequency number of types of plants, *R*^2^ = 0.0065). There was no difference in alpha diversity between the two groups (Faith’s phylogenetic diversity [PD], Shannon diversity, nor observed operational taxonomic unit [OTU] richness; Fig. S1B) and also no difference when groups were stratified by consumption frequency (Table S2).

10.1128/mSystems.00901-19.8 TABLE S2 Alpha diversity comparisons (Kruskal-Wallis) between different frequencies of fermented plant consumption. Download Table S2, PDF file, 0.02 MB. Copyright © 2020 Taylor et al. 2020 Taylor et al. This content is distributed under the terms of the Creative Commons Attribution 4.0 International license.

Next, we used Songbird (27) to identify specific microbes that were associated with consumers or nonconsumers. Songbird is a compositionally aware differential abundance method which provides rankings of features (suboperational taxonomic units [sOTUs]) based on their log fold change with respect to covariates of interest. In this case, the formula we used described whether the subject consumed fermented plants or not. We selected the 20 highest (“set 1,” Table S3)- and 20 lowest (“set 2,” Table S3)-ranked sOTUs associated with fermented plant consumption and used Qurro (28) to compute the log ratio of these sets of taxa (Fig. S1C). Comparing the ratios of taxa in this way mitigates bias from the unknown total microbial load in each sample, and taking the log of this ratio gives equal weight to relative increases and decreases of taxa (27). Evaluation of the Songbird model for fermented plant consumption against a baseline model obtained a *Q*^2^ value of −5.4249, suggesting possible overfitting related to the subtlety of the differences between fermented plant consumption groups. In order to verify the log ratios chosen by Songbird ranks, we performed a permutation test by taking 1,000 random permutations of log ratios with 20 nonoverlapping features in the numerator and denominator. The rank order, compared to the random permutation, was 16, corresponding to a *P* value of 0.0159 (Fig. S2A), suggesting that the log ratio based on the Songbird ranks is nonrandom. We found that consumers have a significantly higher log ratio of set 1 to set 2 than nonconsumers (*t* test, *P* = 0.00065, *t* = 3.6367), suggesting that they are associated with *Bacteroides* spp., *Pseudomonas* spp., *Dorea* spp., *Lachnospiraceae*, *Prevotella* spp., Alistipes putredinis, *Oscillospira* spp., *Enterobacteriaceae*, *Fusobacterium* spp., *Actinomyces* spp., *Achromobacter* spp., Clostridium clostridioforme, Faecalibacterium prausnitzii, Bacteroides uniformis, *Clostridiales*, and *Delftia* spp.

10.1128/mSystems.00901-19.2 FIG S2 Validation of log ratio usage for differentially abundant microbe selections (Songbird) and microbe-metabolite cooccurrence interpretation (mmvec). (A and B) Distribution of the *t* test statistical values obtained for the comparison of consumer versus nonconsumer based on the log ratios of each of 1,000 random microbe selections. One caveat to this type of permutation is the dependence caused by the reuse of features in the generation of the null distribution, with only 3,100 and 153 features employed for the 16S and metagenomic data sets, respectively. (C) Relationship between the location of the metagenomic microbial features along the first axis of the mmvec biplot (Fig. 3c) and their differential abundance ranks (low values = most associated with consumers). Pearson’s correlation *r* score and *P* value are indicated for 249 microbes, with CLA producers colored by taxon. Download FIG S2, PDF file, 0.5 MB. Copyright © 2020 Taylor et al. 2020 Taylor et al. This content is distributed under the terms of the Creative Commons Attribution 4.0 International license.

10.1128/mSystems.00901-19.9 TABLE S3 Sets of taxa used in Songbird analyses. Download Table S3, PDF file, 0.1 MB. Copyright © 2020 Taylor et al. 2020 Taylor et al. This content is distributed under the terms of the Creative Commons Attribution 4.0 International license.

### Gut microbiome composition in frequent and rare fermented food consumers.

One hundred fifteen participants were recruited for a longitudinal study in order to assess the gut microbiome over time and at a finer resolution by using untargeted mass spectrometry in addition to 16S rRNA sequencing (Fig. 1a). We targeted participants who self-identified as frequent consumers or very rare consumers. Consumers were identified using the same definition as in the cross-sectional cohort: consumers ate fermented plants “daily,” “regularly (3 to 5 times/week),” or “occasionally (1 to 2 times/week)”; nonconsumers ate fermented plants “rarely (less than once/week)” or “never” (Fig. S3). The longitudinal cohort was designed to have a higher proportion of consumers who reported eating fermented plants “daily” and “regularly” versus “occasionally” than the cross-sectional cohort (Fig. S4). Similarly, the nonconsumer group in the longitudinal cohort had a higher proportion of participants who reported eating them “never” and “rarely” (Fig. S4) than did nonconsumers in the cross-sectional study.

10.1128/mSystems.00901-19.3 FIG S3 Demographic and selected dietary data for longitudinal cohort samples. (A) Fermented plant consumption frequency within the longitudinal cohort subjects. (B) The other types of fermented food that consumers and nonconsumers reported eating, aside from fermented plants. (C to F) Proportion of subjects in the longitudinal cohort between consumers and nonconsumers, described by sex (chi-square test = 1.31, *P* value = 2.53e−01), age category (chi-square test = 23.85, *P* value = 2.68e−05), number of types of plants consumed (chi-square test = 19.61, *P* value = 5.97e−04), and BMI category (chi-square test = 0.22, *P* value = 8.97e−01; “underweight” subjects were removed in this comparison because there are no underweight nonconsumers in the longitudinal cohort). The total number of individuals is displayed on each bar. Download FIG S3, PDF file, 1 MB. Copyright © 2020 Taylor et al. 2020 Taylor et al. This content is distributed under the terms of the Creative Commons Attribution 4.0 International license.

10.1128/mSystems.00901-19.4 FIG S4 Proportion of fermented plant consumption in the (single-time-point) longitudinal and cross-sectional cohorts. Proportion of subjects within the longitudinal versus the cross-sectional cohorts who consume fermented plants at frequencies of never, rarely, occasionally, regularly, and daily. Download FIG S4, PDF file, 0.5 MB. Copyright © 2020 Taylor et al. 2020 Taylor et al. This content is distributed under the terms of the Creative Commons Attribution 4.0 International license.

A separate fermented food questionnaire was provided to these 115 participants to characterize additional types of fermented food consumed and to evaluate the proxy of fermented plant consumption for general fermented food consumption. Briefly, the major fermented foods consumed were beer, kimchi, kombucha, pickled vegetables, sauerkraut, and yogurt. More consumers reported eating fermented foods than did nonconsumers (Fig. S3B). Only 7.0% of participants (8/115) who stated that they never consumed fermented plants reported consuming another type of fermented food. Of these eight participants, two reported that they consumed wine or beer; one participant reported consuming yogurt, cider, wine, and beer; and five participants reported consuming unspecified fermented foods. We also observed that fermented plant consumers more frequently ate fermented dairy products (yogurt, sour cream/crème fraiche, kefir milk, and cottage cheese) than did nonconsumers (Fig. S3B). Therefore, we further identified them as “fermented food consumers,” in contrast to the cross-sectional cohort.

Within the 16S data, we did not observe a difference in alpha diversity (Shannon’s index [29]) and Faith’s phylogenetic diversity (30) between consumers and nonconsumers (Fig. S1B). We further applied a sparse functional principal-component analysis (31), which explicitly factors in the longitudinal component, and did not observe a significant difference in alpha diversity (Shannon’s index, Wilcoxon *P* = 0.20), suggesting that the stability of alpha diversity in the microbiome over 4 weeks is consistent for consumers and nonconsumers.

**FIG 2:**
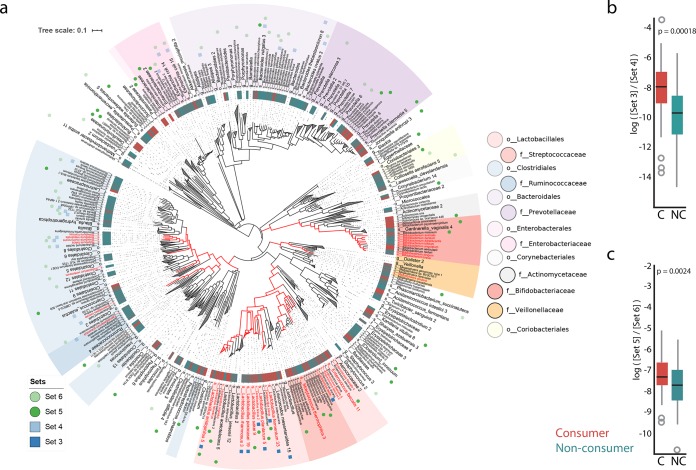
Phylogenetic and log ratio differences between consumers and nonconsumers in gut metagenomes. (a) Phylogenetic diversity captured in the metagenomes. The species taxon hits to the rep82 database were mapped to the species taxa of the Web of Life database (74) (97.8% mapped) in order to represent the phylogenetic distances computed from full genomes. The species known to produce CLA are indicated in red font. The species sets used for log ratio calculations are labeled using opaque (numerators) and transparent (denominators) colors. Set 3 is composed of microbes identified from reference 6, and set 4 contains the most prevalent microbes across all samples (blue squares). Set 5 and set 6 are derived from Songbird (green circles). (b) Consumers have a significantly higher ratio of set 3 to set 4 (*t* test, *P* value = 0.0001838, *t* = 3.9386, Cohen’s *D* = 0.851). (c) The log ratio of set 5 to set 6 is significantly different (box plot, *t* test *P* = 0.0024, *t* = 3.15, Cohen’s *D* = 0.692). The lists of microbes in each set are available in Table S3.

A subset of 100 samples were sequenced by shotgun metagenomics to provide a finer resolution of the taxonomic differences between the two groups. First, we verified whether the gut microbiota of self-reported fermented food consumers was associated with fermented food-associated species. We computed a log ratio using Qurro (28) of fermented food-associated taxa according to the work of Marco et al. (6) (“set 3,” Table S3) compared to a set of taxa that were present across all samples (“set 4,” Table S3) (Fig. 2b). Eight species were detected in our data set and were used to compute this log ratio: Lactobacillus acidophilus, Lactobacillus brevis, Lactobacillus fermentum, Lactococcus lactis, Leuconostoc mesenteroides, Lactobacillus paracasei, Lactobacillus plantarum, and Lactobacillus rhamnosus (Fig. 2a). We found that consumers had a significantly higher log ratio of set 3 to set 4 than nonconsumers (*t* test, *P* value = 0.0001838, *t* = 3.9386, Cohen’s *D* = 0.851), suggesting that consumers were associated with some taxa derived from fermented foods.

We then used Songbird (27) to test whether there was a broader set of microbial features associated with consumers or nonconsumers. We selected the 40 highest-ranked (“set 5,” Table S3) and 40 lowest-ranked (“set 6,” Table S3) microbes associated with fermented plant consumption and used Qurro to compute the log ratio of these sets of taxa (Fig. 2c); these were the smallest sets of features that provided meaningful differences between consumers and nonconsumers. Again, because evaluation of the Songbird models for fermented plant consumption against a baseline model suggested overfitting (*Q*^2^ value of −0.12), we further verified the log ratios chosen by Songbird ranks by performing a permutation test of taking 1,000 random permutations of log ratios with 20 nonoverlapping features in the numerator and denominator. The rank order, compared to the random permutation, was 2, corresponding to a *P* value of 0.0019 (Fig. S2B), suggesting that the log ratio based on the Songbird ranks is nonrandom. This analysis at the species level showed that consumers have a significantly higher log ratio of set 5 to set 6 than nonconsumers (*t* test *P* = 0.0024, *t* = 3.15, Cohen’s *D* = 0.692).

Several microbes of relevance to fermented foods were also associated with consumers, including Lactobacillus acidophilus, Lactobacillus brevis, Lactobacillus kefiranofaciens, Lactobacillus parabuchneri, Lactobacillus helveticus, and Lactobacillus sakei (6, 32–35) (Fig. 2a). Consumers were also associated with several other microbes unrelated to fermented foods, including Streptococcus dysgalactiae, Prevotella melaninogenica, Enorma massiliensis, Prevotella multiformis, Enterococcus cecorum, and Bacteroides paurosaccharolyticus. The microbes that distinguish consumers and nonconsumers in the cross-sectional and longitudinal data sets may not fully overlap because the longitudinal cohort was intentionally composed of participants in the more “extreme” ends of consumption (individuals who consume “daily” and “regularly” versus individuals who “never” consume fermented plants), because the cohorts were analyzed using different sequencing methods (16S versus metagenomics), or because of a combination of these aspects.

### The functional profile of the gut microbiome differs with consumption of fermented food.

To assess the functional profile of the gut microbiome of specifically recruited fermented food consumers and nonconsumers, we performed untargeted HPLC-MS/MS analysis on all longitudinal samples (115 subjects, 417 samples, with up to 4 samples per subject, collected weekly for 4 weeks) (Fig. 1a). We explored the longitudinal stability using both the 16S and mass spectrometry data and found that the taxa and metabolites remained stable (Spearman’s rho ranging from 0.42 to 0.68; *P* < 0.001) between time points within both consumers and nonconsumers (Fig. S5). The correlation coefficients for metabolites tended to be lower than for the taxa, suggesting more volatility in the observed metabolic features. This is expected since the metabolome is driven in large part by the diet, which changes day to day.

10.1128/mSystems.00901-19.5 FIG S5 Longitudinal stability of microbes and metabolites (correlations were assessed by Spearman rho). The first and third columns are based on the Songbird 16S results and metabolomics results, respectively, for all longitudinal samples. The second and fourth columns consider consumers and nonconsumers separately. The first column shows the log ratios of the sets of taxa identified using Songbird on the 16S data between time points. 16S signatures, within participants and within data type, appear consistent over time based on Spearman’s rho. The second column plots these log ratios for consumers and nonconsumers separately. The third column shows the log ratios of the sets of annotated mass spectrometry features identified using Songbird between time points. The fourth column plots these log ratios for consumers and nonconsumers separately. Download FIG S5, PDF file, 1.1 MB. Copyright © 2020 Taylor et al. 2020 Taylor et al. This content is distributed under the terms of the Creative Commons Attribution 4.0 International license.

**FIG 3:**
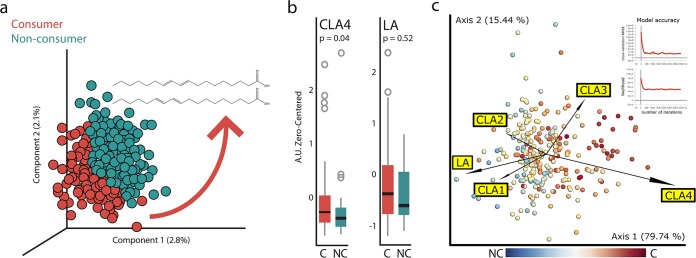
Conjugated linoleic acid is significantly higher in consumers than nonconsumers. (a) Partial least squares discriminatory analysis (PLS-DA) of untargeted mass spectrometry data identified CLA as one of the discriminating features in fermented food consumer samples. (b) Zero-centered counts of MS1 features annotated with a CLA isomer (designated “CLA4”) and with the unconjugated linoleic acid (LA) between consumers and nonconsumers. “CLA4” is enriched in the consumer group (Wilcoxon test, *P* value = 0.04) but not LA (Wilcoxon test, *P* value = 0.52). (c) Integrative analysis of metagenomics and mass spectrometry data sets using mmvec. Genome features (dots) are labeled according to their strength of change with respect to fermented food consumption (red is associated with consumption, blue with nonconsumption). The metabolites are represented by arrows indicating their cooccurrences with the genomes.

Using partial least squares discriminatory analysis (PLS-DA), we found that notable differences exist between consumers and nonconsumers when all time points were taken into account (Fig. 3a; Fig. S6A). The majority of the top discriminating features appeared to be lipids, several of which have broad natural distributions and thus are likely common. In particular, one compound was identified as octadecadienoic acid and then determined specifically to be an isomer of conjugated linoleic acid (CLA). At a single time point, we found that this isomer of CLA (designated “CLA4”; the exact configuration is unknown) was enriched in consumers (Wilcoxon test, *P* value = 0.04) whereas the unconjugated linoleic acid (LA) was not significantly different between the two groups (Wilcoxon test, *P* value = 0.52) (Fig. 3b). As CLA has also been found as one of the discriminating features in samples from subjects who consume a large number of types of plants (25), it might suggest that the difference between consumers and nonconsumers could be partly explained by the number of types of plants consumed. However, in this study CLA abundances were not significantly different between the two extreme groups of types of plant consumption: fewer than 10 types of plants versus more than 30 types of plants (Wilcoxon rank sum test, *P* value = 0.98). From the food frequency questionnaire, we found that dietary consumption of total LA (18:2 n-6; g/day) and total CLA (g/day) did not differ significantly between consumers and nonconsumers (Fig. S6B), suggesting that the elevated levels of CLA in the fecal samples of consumers are likely derived from an endogenous process or microbial origin.

10.1128/mSystems.00901-19.6 FIG S6 (A) Variable importance in the projection (VIP) scores of the top 15 features discriminating between consumers and nonconsumers. Abundance across categories is represented by color marking on the right (green is low, red is high). The values under “feature” refer to the detected *m/z*. The putative annotations from GNPS library search of the top 10 features discriminating consumer and nonconsumer categories are included next to the features. (B) CLA and LA consumption from the Food frequency questionnaire does not differ between consumers and nonconsumers. Comparison of dietary CLA and LA in consumers and nonconsumers (Wilcoxon rank sum test with Bonferroni-Hochberg correction for multiple testing) for both cross-sectional and longitudinal data sets. Sample sizes indicated on the right-hand side. Test *P* values are indicated under each panel. Download FIG S6, PDF file, 0.8 MB. Copyright © 2020 Taylor et al. 2020 Taylor et al. This content is distributed under the terms of the Creative Commons Attribution 4.0 International license.

A total of 79 samples were analyzed using both metagenomic sequencing and mass spectrometry (Fig. 1a). We used mmvec (36) to integrate these data to assess cooccurrence patterns between genomic features (species) and the LA and CLA metabolites. We found that “CLA4,” which was significantly enriched in consumers, cooccurs with the species (previously identified using Songbird) that were most strongly associated with consumers. Additionally, we found that linoleic acid (LA) cooccurs with the microbes that are most strongly associated with nonconsumers (Fig. 3c). Of the top 50 taxa that had the highest probability of cooccurring with “CLA,” 14 are known CLA producers. These include Eubacterium rectale, Faecalibacterium prausnitzii, Eubacterium siraeum, Eubacterium hallii, Bifidobacterium adolescentis, and genera *Roseburia*, *Anaerostipes*, *Eubacterium*, *Ruminococcus*, and *Clostridium* (Table S4) (37–40). Forty-eight out of these top 50 taxa were more abundant in consumers than nonconsumers (Table S4).

10.1128/mSystems.00901-19.10 TABLE S4 mmvec, species and CLA4 cooccurrence. Species (rep82 features) are listed in the column “Feature” and are sorted by “mmvecRank,” the ranked conditional probabilities of cooccurrence between rep82 features and “CLA4.” Species known to produce CLA appear in the column “CLA-producer.” The column “mmvecPC1” contains the associated taxon features and their PC1 axis values from Fig. 3c. The last two columns display the numbers of consumers and nonconsumers who have the feature. Download Table S4, PDF file, 0.1 MB. Copyright © 2020 Taylor et al. 2020 Taylor et al. This content is distributed under the terms of the Creative Commons Attribution 4.0 International license.

## DISCUSSION

In this study, we explored the gut microbiome of fermented plant consumers and nonconsumers in the American Gut Project (25), an extensive collection of sample contributions from tens of thousands of citizen scientists. Gut microbiome profiles, but not overall microbial diversity, differed slightly between the groups, suggesting that small but systematic compositional differences may occur based on a dietary choice to consume fermented plants. In a concurrent targeted longitudinal study, we found that fermented-food related taxa as well as a putatively health-associated molecule were associated with consumers. Several microbes that were found to be associated with fermented consumers include microbes known to be derived from fermented foods, including fermented milk products (Lactobacillus acidophilus [6], Lactobacillus brevis [6], Lactobacillus kefiranofaciens [32], Lactobacillus parabuchneri [33], and Lactobacillus helveticus [34]) and fermented meat (Lactobacillus sakei [35]). This is consistent with other metagenomic studies from population-based cohorts that detected species related to starters such as Leuconostoc mesenteroides and Lactococcus lactis in subjects who consumed a specific fermented milk product (buttermilk) in the Dutch cohort Lifeline DEEP (20).

Analysis of the metabolomics data using PLS-DA found that shifts in lipid metabolism were associated with consumption of fermented plants, since the majority of the top discriminating metabolites appeared to be lipids. Of those that could be identified, CLA was particularly notable. The abundance of the CLA isomer “CLA4”is significantly increased in consumers over nonconsumers. CLA is known to be produced during ruminal bacterial fermentation and impacts the fatty acid composition of meat and dairy products from ruminants that represent the major dietary sources of CLA in humans (40). Due to its possible health benefits (41, 42), CLA is also often consumed as a nutritional supplement. However, CLA fecal recovery did not correlate with dietary CLA intake derived mainly from meat, full-fat dairy, and egg sources as determined by the food frequency questionnaire (FFQ). Moreover, dietary consumption of total CLA (grams/day) did not differ between consumers and nonconsumers. Thus, it is possible that CLA is being produced by resident or transient bacteria derived from fermented foods.

Indeed, diet-related bacteria, such as LAB, bifidobacteria, and propionibacteria, have been shown previously to produce CLA (39). Intestinal bacteria belonging to the families *Lachnospiraceae* and *Ruminococcaceae* have also been shown to metabolize LA into products that can be precursors of CLA (37), and two of these *Lachnospiraceae* were also found to be associated with consumers. The order *Lactobacillales* includes the largest diversity of previously reported CLA producers, and notably, seven out of the eight species previously identified as associated with fermented foods (set 3) are CLA-producing *Lactobacillus* species that we found to be associated with fermented food consumers: L. acidophilus, L. brevis, *L. fermentum*, L. helveticus, *L. paracasei*, *L. plantarum,* and *L. sakei* (for reviews, see references 38 to 40, 43). However, increased CLA in consumers cannot be fully attributed to production by fermented food-associated bacteria. For example, some members of the order *Clostridiales* previously reported to produce CLA in human feces (including four *Roseburia* species: R. inulinivorans, R. hominis, R. intestinalis, and R. faecis [37]) were found to be associated with nonconsumers, along with Anaerostipes caccae, Eubacterium ventriosum (L2-12), and Faecalibacterium prausnitzii, which are also known to metabolize LA.

We detected seven *Bifidobacterium* species previously reported to produce CLA using LA as a precursor (38, 39), including Bifidobacterium animalis, B. longum (44), and *B. breve*, which has been considered for CLA enrichment in commercial foods such as yogurt due to its CLA-producing ability (45). Yet, none of these were found to be associated with the fermented food consumers. Rather, two other *Bifidobacterium* species not known to produce CLA (B. aesculapii and B. reuteri) were found to be associated with fermented food consumers, with *B. reuteri* growth actually inhibited at high concentrations of LA precursor (46). Moreover, of the top 50 taxa that were identified as having the highest probability of cooccurring with “CLA4,” only 14 were known CLA producers (see Table S4 in the supplemental material). Future investigation into metabolic pathways in larger data sets would allow the identification of species that explain the higher abundance of “CLA4” in consumers than in nonconsumers.

This is to our knowledge the largest study of the association between fermented food (specifically, fermented plant) consumption and the human gut microbiome, with nearly 7,000 individuals at one time point and over 100 individuals across 4 weeks of sampling. We took a multi-omic approach—a combination of 16S rRNA sequencing, shotgun metagenomics, and mass spectrometry—coupled with state-of-the-art tools to evaluate the data. We find that the consumption of fermented plants and, more broadly, fermented foods is associated with quite subtle microbiome variation in healthy individuals. While this explorative study provides the foundation for more-directed research, such as randomized placebo-controlled studies, it has some limitations, particularly that consumers were categorized according to self-reported frequency of fermented plant consumption. First, self-reported dietary information can be flawed with measurement errors (47). Second, although our data suggest that fermented plant consumption may be a reasonable proxy for consumption of fermented food more generally, they do not explicitly take into account other food types, such as fermented dairy products. Additionally, this study is mostly limited to participants living in the United States, who may consume a lower diversity of fermented foods than populations living in other countries; expanding this study to a wider range of populations would allow us to capture a greater diversity of fermented food types and associated microbial communities. Due to a combination of these factors, we may be underestimating the potential effects of fermented food consumption on the gut microbiome. Yet notably, the recovery of LAB and fermented-food-derived microbes in the stool of self-reported consumers suggests that data from stool may be used to help verify the reliability of self-reported dietary information. It would therefore be of great relevance to evaluate not only the associations between specific types of food and the microbiome but also our ability to detect consumption of specific fermented foods in future studies.

## MATERIALS AND METHODS

### Participant recruitment, sample processing, and sample selection.

This research was performed in accordance with the University of Colorado Boulder’s Institutional Review Board protocol number 12-0582 and the University of California San Diego’s Human Research Protection Program protocol number 141853. In order to investigate the effect of fermented plant and food consumption on the gut microbiome, a retrospective analysis was performed on the American Gut Project data set (25). An additional cohort of 115 subjects was recruited to explore the effect of fermented food consumption or nonconsumption over a period of 4 weeks; the samples from the longitudinal cohort were processed and sequenced in accordance with AGP protocol and integrated into the AGP data set. The time point with the highest read count from each of the 115 recruited individuals was added to the concurrent cross-sectional assessment. The longitudinal cohort also responded to a specific fermented food questionnaire.

The entire AGP data set was subset using the metadata version accessed 8 August 2019 for stool samples from adult participants (age >19 and <70 years) who answered the “fermented plant frequency” question from the AGP questionnaire. Participants were excluded if they took antibiotics in the last year or if they had outlier values for their body mass index (<15 or >50), height (<48 cm or >210 cm), or weight (<2.5 kg or >200 kg). If biological replicates were present, the replicate with the lower number of reads was removed (with the exception of the 115 participants who constitute the longitudinal cohort). Based on the AGP questionnaire, participants were considered consumers if they reported “daily,” “frequent” and “occasional” fermented plant consumption (i.e., >1 to 2 times per week) and nonconsumers if they reported “rarely” and “never.”

### Diet quality and intake assessment.

Overall diet quality was assessed by the Healthy Eating Index 2010 (HEI-2010) as described elsewhere (48). Briefly, the HEI-2010 is a valid, reliable measure of diet quality that assesses how an individual’s diet pattern adheres to the 2010 to 2015 Dietary Guidelines for Americans (DGA). HEI-2010 includes 12 dietary components, nine of which are classified as “adequacy” components that should be included regularly in the diet (total fruit, whole fruit, total vegetables, greens and beans, whole grains, dairy, total protein foods, seafood and plant proteins, and fatty acids), and 3 “moderation” components (refined grains, sodium, and empty calories) that should be limited in the diet. Individual dietary components are scored from 0 to 5, 10, or 20 points with maximum points indicating higher consumption of adequacy components and lower consumption of moderation components. Total HEI-2010 scores (range: 0 to 100) were calculated as the sum of the 12 components with a higher total score indicating better/optimal diet quality and greater adherence to the DGA. HEI-2010 scores, as well as total energy, carbohydrate, fat, protein, and fiber intake, were calculated from individuals in the AGP cohort who completed the VioScreen food frequency questionnaire (FFQ). We compared the total HEI score and mean nutrient intakes between consumers and nonconsumers using the Mann-Whitney U test.

Daily total consumption of CLA and LA (grams/day) was estimated from the VioScreen FFQ reports. Total CLA consumption was deduced from the following food sources: beef and other meat such as fish and turkey, full-fat dairy products (e.g., milk, butter, cheese, and yogurt), and eggs. Total LA consumption was obtained from the following reported foods: vegetable oil (e.g., canola and olive), salad dressings containing vegetable oils, butter, eggs, meat (beef, chicken, turkey, and pork), potatoes (e.g., French fries/fried white potatoes, and potato chips), nuts, nut butters and seeds, mixed Mexican dishes, and meat dishes such as stews and casseroles.

### 16S rRNA gene sequencing.

DNA extraction and 16S rRNA amplicon sequencing were done using Earth Microbiome Project (EMP) standard protocols (http://www.earthmicrobiome.org/protocols-and-standards/16s). DNA was extracted with the Qiagen MagAttract PowerSoil DNA kit as previously described (49). Amplicon PCR was performed on the V4 region of the 16S rRNA gene using the primer pair 515f-806r with Golay error-correcting barcodes on the reverse primer. Amplicons were barcoded and pooled in equal concentrations for sequencing. The amplicon pool was purified with the Mo Bio UltraClean PCR cleanup kit and sequenced on the Illumina MiSeq sequencing platform. Based on the filtering noted above, a feature table representing the 16S V4 rRNA gene sequence data was obtained from Qiita (50) using redbiom (51) from the Deblur-Illumina-16S-V4-150nt-780653 context. This table was composed of 8,513 samples. Prior to extraction from Qiita, the AGP data had been trimmed to 150 bases and processed using Deblur v1.0.4 (52) using the Qiita default parameters (i.e., setting –min-reads 1) to generate sOTUs. Technical replicates of samples were excluded in order to keep only the most-sequenced version of each sample. After previously recognized bloom sequences were removed (53), samples with fewer than 1,500 reads were omitted. Taxonomies for sOTUs were assigned using the sklearn-based taxonomy classifier trained on the Greengenes reference database 13_8 (54) clustered at 99% similarity (feature classifier plug-in of QIIME 2 v2019.1 [55]). The sOTU table was rarefied to a depth of 1,500 sequences/sample to control for sequencing effort (56) and sOTUs totaling 5 reads across samples. The deblurred sequence fragments were inserted into the Greengenes 13_8 phylogenetic tree using SATé-enabled phylogenetic placement (57, 58).

### 16S marker gene data analysis.

QIIME 2 v2019.1 (55) was used to generate pairwise unweighted and weighted UniFrac distances (51, 59). Between-group differences based on these distances were tested using PERMANOVA (60) and permuted *t* tests in QIIME 2. Alpha diversity (Faith’s PD [30], Shannon diversity [29], and observed OTU richness) between consumers and nonconsumers (as a whole and when stratified by consumption frequency) was generated using QIIME 2 (55) and compared with a Kruskal-Wallis test. Wilcoxon signed-rank (61) and Mann-Whitney U tests were used to assess alpha diversity between successive time points within consumers and nonconsumers and within time point between consumers and nonconsumers in the longitudinal cohort, respectively. Songbird v1.0.1 (27) was used to identify feature ranks corresponding to consumers and nonconsumers (parameters: –epochs 5000 –batch-size 5 –learning-rate 1e−4 –min-sample-count 1000 –min-feature-count 0 –num-random-test-examples 10), and Qurro v0.4.0 (28) was used to compute log ratios of these ranked features. *t* tests and Cohen’s *D* were calculated to assess the significance (alpha = 0.05) and effect size of the log ratios. The stability of the participants’ microbiomes was assessed by comparing sample log ratios in consecutive time points, for both the 16S and metabolomic data sets. The 40 highest- and lowest-ranked features were used in order to compute enough log ratios for Spearman’s rank correlation coefficients across all samples and for ordinary least squares regression (Fig. S5).

### LC-MS/MS data acquisition.

The untargeted metabolomics analysis using high-performance liquid chromatography–tandem mass spectrometry (HPLC-MS) was carried out as described previously (25). The chromatography was performed on a Dionex UltiMate 3000 Thermo Fisher Scientific high-performance liquid chromatography system (Thermo Fisher Scientific, Waltham, MA) coupled to a Bruker Impact HD quadrupole time of flight (qTOF) mass spectrometer. The chromatographic separation was carried out on a reverse-phase (RP) Kinetex C_18_ 1.7-μm, 100-Å ultrahigh-performance liquid chromatography (UHPLC) column (50 mm by 2.1 mm) (Phenomenex, Torrance, CA), held at 40°C during analysis. A total of 5 μl of each sample was injected. Mobile phase A was water, and mobile phase B was acetonitrile, both with added 0.1% (vol/vol) formic acid. The solvent gradient table was set as follows: initial mobile phase composition was 5% B for 1 min, increased to 40% B over 1 min and then to 100% B over 6 min, held at 100% B for 1 min, and decreased back to 5% B in 0.1 min, followed by a washout cycle and equilibration for a total analysis time of 13 min. The scanned *m/z* range was 80 to 2,000, the capillary voltage was 4,500 V, the nebulizer gas pressure was 2 × 10^5^ Pa, the drying gas flow rate was 9 liters/min, and the temperature was 200°C. Each full MS scan was followed by tandem MS (MS/MS) using collision-induced dissociation (CID) fragmentation of the seven most abundant ions in the spectrum. For MS/MS, the collision cell collision energy was set at 3 eV and the collision energy was stepped 50%, 75%, 150%, and 200% to obtain optimal fragmentation for differentially sized ions. The scan rate was 3 Hz. An HP-921 lock mass compound was infused during the analysis to carry out postprocessing mass correction. To determine the specific isomer of the annotations for octadecadienoic acid isomers, authentic standards for linoleic acid (LA; Spectrum Laboratory Products, Inc., USA) and conjugated linoleic acid (CLA; mixture of 4 isomers: 9,11 and 10,12 isomers, E and Z) (Sigma-Aldrich, USA) were compared by retention times (RTs) and MS/MS spectra. This brings these annotations to the level 1 identifications (authentic compound was analyzed under identical experimental conditions with orthogonal physical property compared).

### LC-MS/MS data analysis.

The collected data were processed as described in reference 62. Briefly, the feature tables were obtained using MZmine2 (63). The collected HPLC-MS raw data were converted from Bruker’s .d to .mzXML format. The data were then batch processed with the following settings for each step: (i) mass detection, noise level of 1,000, chromatogram builder, minimum time span of 0.01 min, minimum peak height of 3,000, and *m/z* tolerance of 0.1 *m/z* or 20 ppm; (ii) chromatogram deconvolution—baseline cutoff, minimum peak height of 3,000, peak duration range of 0.01 to 3.00 min, and baseline level of 300; (iii) deisotopization—isotopic peak grouper, *m/z* tolerance of 0.1 *m/z* or 20 ppm, RT tolerance of 0.1 min, and maximum charge of 4; (iv) peak alignment—join aligner, *m/z* tolerance of 0.1 *m/z* or 20 ppm, weight for *m/z* 75, weight for RT 25, and RT tolerance of 0.1 min; and (v) peak filtering—peak list raw filter, minimum peak in a row of 3 and minimum peak in an isotope pattern of 2.

The metadata were added into the resulting extracted feature table and used as input for the MetaboAnalyst software (64, 65). The feature tables were filtered with interquantile ranges to remove outliers, and the data were imputed, normalized by the quantile normalization, and autoscaled (mean centering and dividing by the standard deviation for each feature). Partial least-squares discriminant analysis (PLS-DA) was used to explore and visualize variance within data and differences among experimental categories. The CLA and LA metabolite features were identified manually based on GNPS (66) and MZmine 2 (63) processing pipelines (see link below to feature-based molecular networking). The Wilcoxon rank sum test (Mann-Whitney U test) was used to assess the significance of difference between the consumers and nonconsumers for the levels of identified CLA and LA metabolites (alpha = 0.05).

The annotations and visualizations of chemical distributions were explored on GNPS using molecular networking (66) as follows. MS/MS spectra were window filtered by choosing only the top 6 peaks in the 50-Da window throughout the spectrum. The MS spectra were then clustered with a parent mass tolerance of 0.02 Da and an MS/MS fragment ion tolerance of 0.02; consensus spectra that contained fewer than 4 spectra were discarded. The network was created with edges filtered to have a cosine score above 0.65 and more than 5 matched peaks. The edges between two nodes are kept in the network if and only if each of the nodes appears in each other’s respective top 10 most similar nodes. The required library matches were set to have a score above 0.7 and at least 6 matched peaks when searching the spectra in the network against GNPS spectral libraries. All resulting annotations are at level 2/3 according to the proposed minimum standards in metabolomics (67). The GNPS results are located at https://gnps.ucsd.edu/ProteoSAFe/status.jsp?task=420a545b5b164d10a20f62c0ec0ce7e7. Feature-based molecular network (68) results can be found at https://gnps.ucsd.edu/ProteoSAFe/status.jsp?task=9ce1517e83a94d9a8cd9d79f3e16eea0. The CLA and LA metabolite features were initially identified based on GNPS library search (66), and then their annotation was further confirmed via use of authentic standards. The Wilcoxon rank sum test was used to assess the significance of difference between the consumers and nonconsumers for the levels of identified CLA and LA metabolites (alpha = 0.05).

### Metagenomic sequencing.

Extracted DNA was quantified with the PicoGreen double-stranded DNA (dsDNA) assay kit, and 5 ng of input, or a maximum of 3.5 μl, genomic DNA (gDNA) was used in a 1:10 miniaturized Kapa HyperPlus protocol. Per-sample libraries were quantified and pooled at equal nanomolar concentrations. The pooled library was cleaned with the QIAquick PCR purification kit and size selected for fragments between 300 and 700 bp on the Sage Science PippinHT. The pooled library was sequenced as a paired-end 150-cycle run on an Illumina HiSeq2500 v2 in Rapid Run mode at the UCSD IGM Genomics Center, with a target depth of ca. 20 million reads per sample. The sequencing adapter and short reads were first removed using Atropos v1.1.21 (-q 15 –minimum-length 100 –pair-filter any) as well as reads aligning to the human genome using bowtie2 (–very-sensitive). The pass-filter reads were then concatenated per sample, excluding 1 biological duplicate and 8 samples from participants exposed to antibiotics, in order to obtain 91 pairs of fastq files.

### Metagenomic data analysis.

On each separate sample fastq file, paired-end reads were merged using FLASH v1.2.11 (69) and then processed for taxonomic profiling using SHOGUN v1.0.6 (70) with Bowtie 2 v2.3.4.3 (71) to align reads to the 85,626 prokaryotic genomes covering 12,977 species from the NCBI RefSeq database release 82 (72). The read counts for the genome features identified in each sample were merged into one genome-per-sample table that was then filtered to keep genomes with a per-sample relative mapped read abundance of at least 0.01%. The features labeled at the subspecies level were sum collapsed at the species level; taxonomy was used as a proxy for a phylogeny. As with the 16S cross-sectional data, Songbird (Songbird v1.0.1 [27]) was used for regression modeling on our binary fermented consumption variable to identify features associated with consumption and nonconsumption (parameters as above). Qurro v0.4.0 (28) was used to compute log ratios of these ranked features. *t* tests and Cohen’s *D* were calculated to assess the significance (alpha = 0.05) and effect size of the log ratios.

### Multi-omics data analysis.

In order to identify microbial features associated with fermented food consumption and the metabolites they might be producing, we measured probabilities of cooccurrence between observed species (based on metagenomic data) and either all metabolites, or a set of five linoleic and isomers of conjugated linoleic acids discernible in the data (as informed by the metabolomic analysis). For this analysis, we used mmvec v1.0.2 (36), a neural network solution inspired from natural language processing, to build a log-transformed conditional probability matrix from each cross-omics feature pair and apply singular value decomposition in order to represent cooccurrence in the form of biplots. We chose the model where accuracy was highest for different initialization conditions for the gradient descent algorithm (–batch-size of 1,000, 2,500, and 5,000 and –learn-rate of 1e−4 and 1e−5), with low cross-validation error and model likelihood. To evaluate the fitness of the mmvec microbe-metabolite interactions, we compared the latent representation to the observed Songbird differentials. The relationship between the microbial first principal component learned from mmvec and the log fold change of the microbes between fermented food consumption was significantly negatively correlated (Pearson’s *r* = −0.651, *P* = 4.63e−22, *n* = 249 microbes; Fig. S2C), suggesting that the mmvec microbe-metabolite relationship to fermented food consumption is a valid comparison. We used EMPeror v2019.1.0 (73) to visualize feature-feature biplots along with overlying genomedifferential abundance ranks for our fermented food consumption model.

### Data availability.

The data generated in this study are available publicly in Qiita under the study ID 10317. Sequence data associated with this study can be found under EBI accession ERP012803. The metabolomics analysis is available at https://gnps.ucsd.edu/ProteoSAFe/status.jsp?task=420a545b5b164d10a20f62c0ec0ce7e7 (classical molecular networking) and https://gnps.ucsd.edu/ProteoSAFe/status.jsp?task=9ce1517e83a94d9a8cd9d79f3e16eea0 (feature-based molecular networking). All of the raw data are publicly available at the UCSD Center for Computational Mass Spectrometry (data set ID MassIVE MSV000081171, https://massive.ucsd.edu/ProteoSAFe/dataset.jsp?task=9996246aab414427a80bb5a451ec3c3d).
