# Supplementary figures and images for: Consumption of Fermented Foods Is Associated with Systematic Differences in the Gut Microbiome and Metabolome
Source: mSystems. 2020 Mar 17;5(2):e00901-19. doi: 10.1128/mSystems.00901-19 (PMC7380580; doi:10.1128/mSystems.00901-19)

A

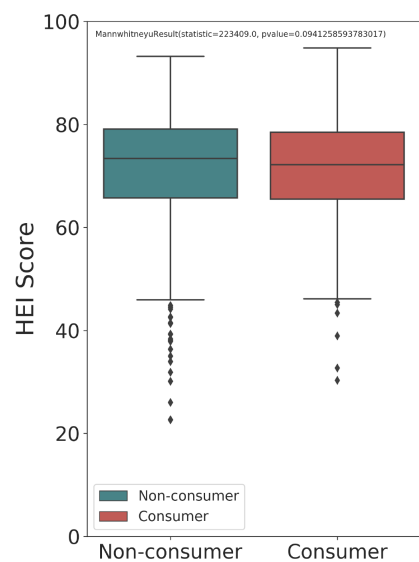

B

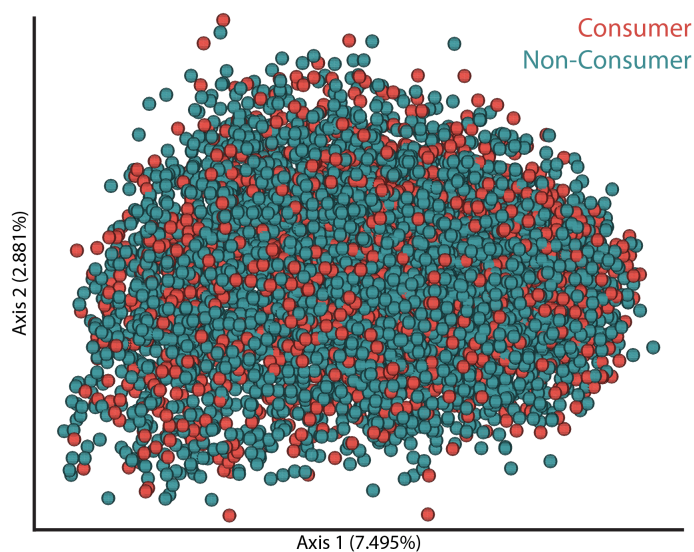

C

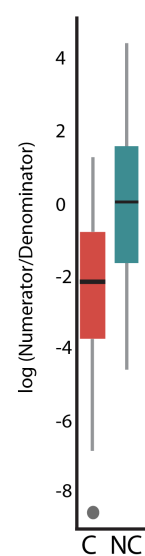

Supplement: FIG S1 [file mSystems.00901-19-sf001.pdf]

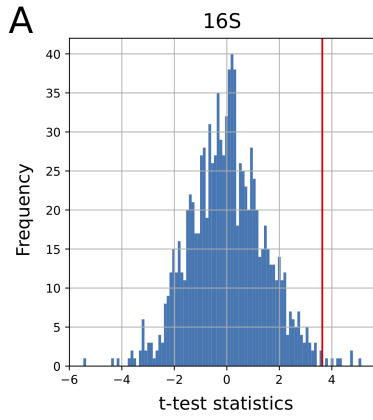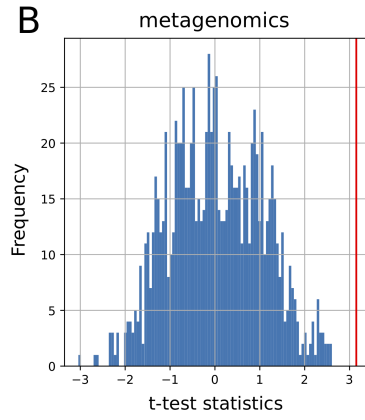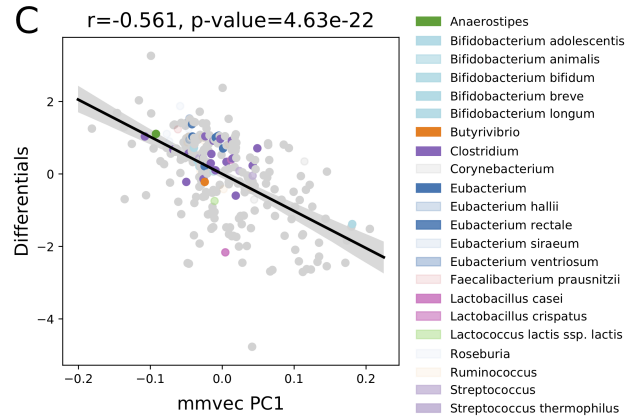

Supplement: FIG S2 [file mSystems.00901-19-sf002.pdf]

# Demographic Data for Longitudinal Cohort Samples

A

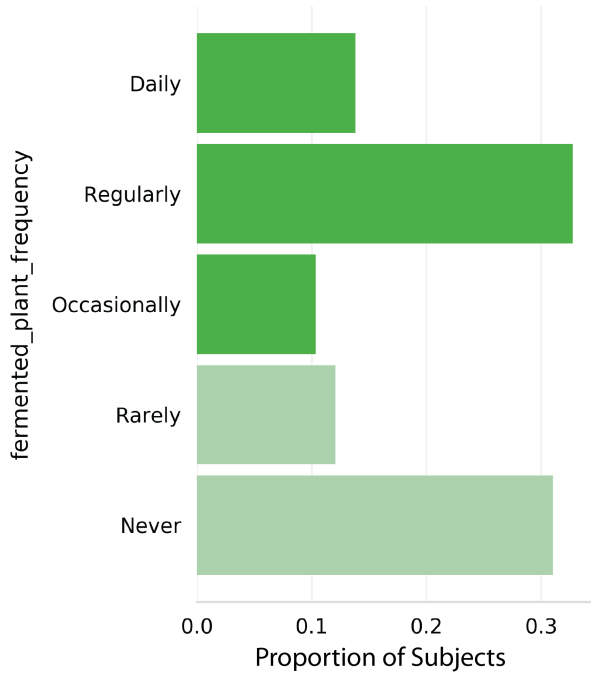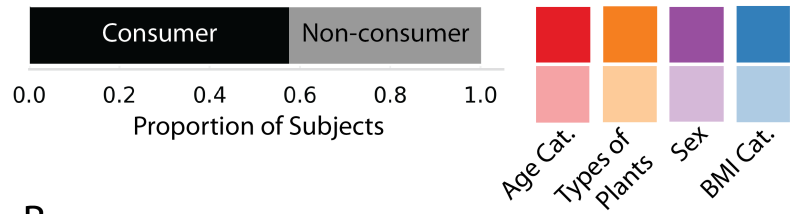

B

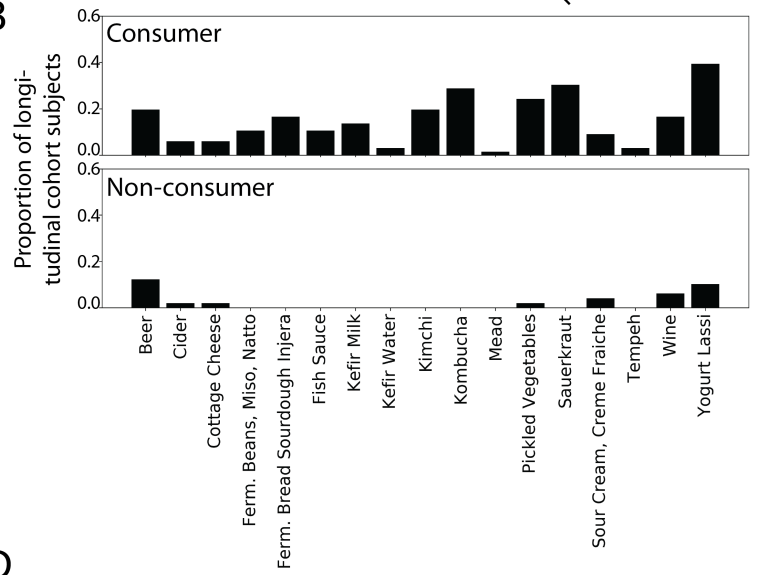

C

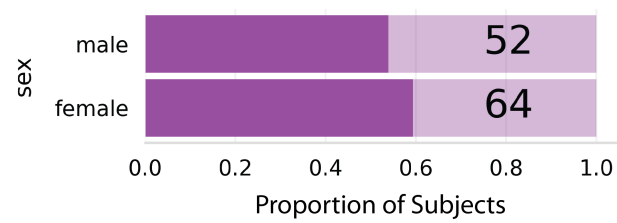

D

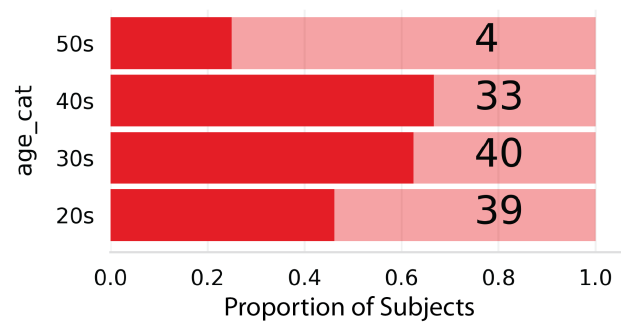

E

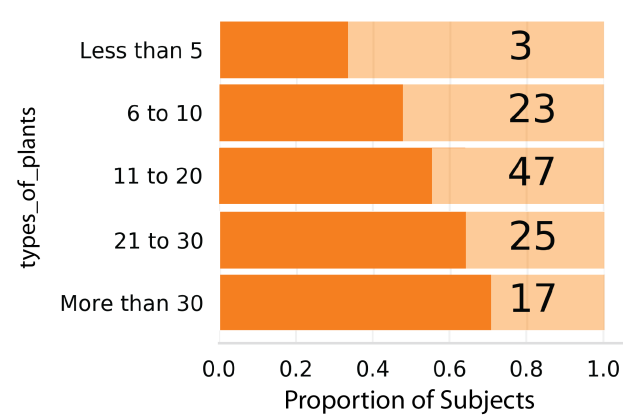

F

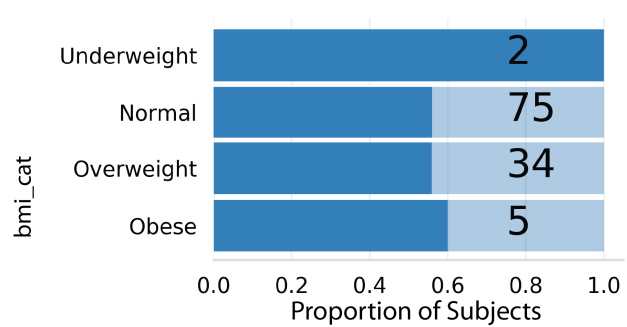

Supplement: FIG S3 [file mSystems.00901-19-sf003.pdf]

## Fermented Plant Frequency: Longitudinal and Cross-Sectional Cohorts

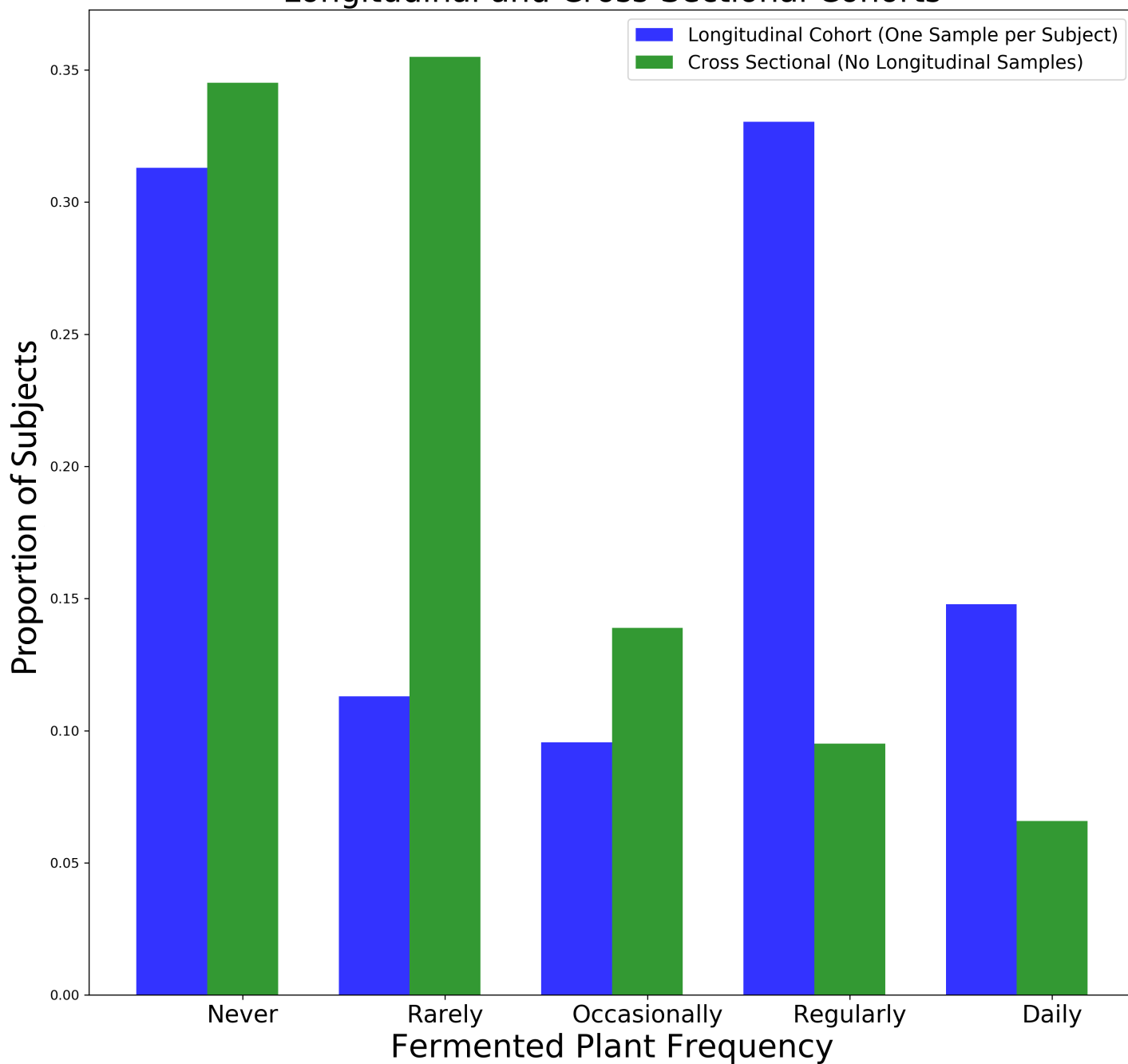

Supplement: FIG S4 [file mSystems.00901-19-sf004.pdf]

16S

● Consumer ● Non-consumer

Metabolomics

t0 vs. t1

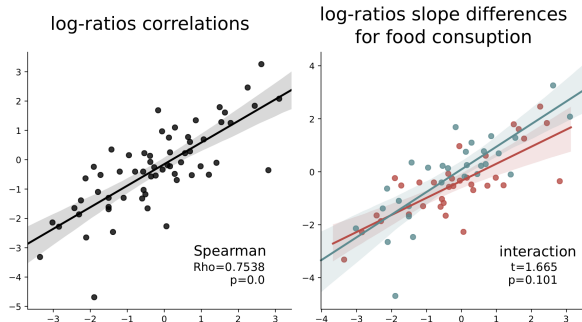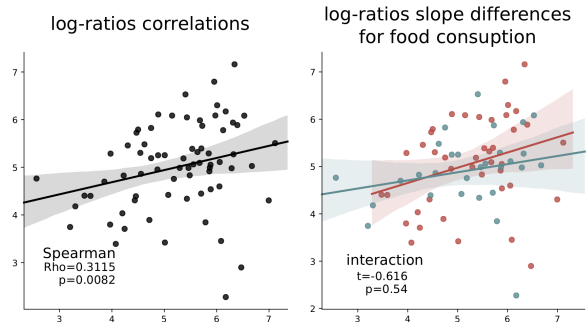

t1 vs. t2

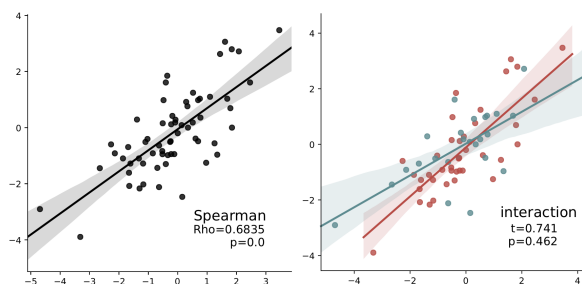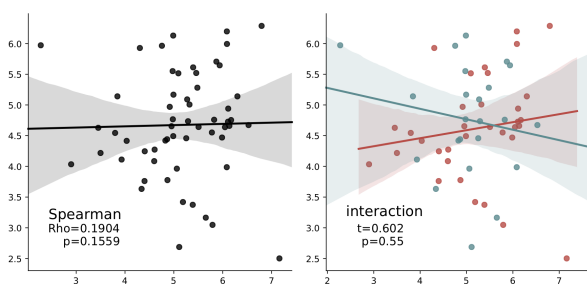

t2 vs. t3

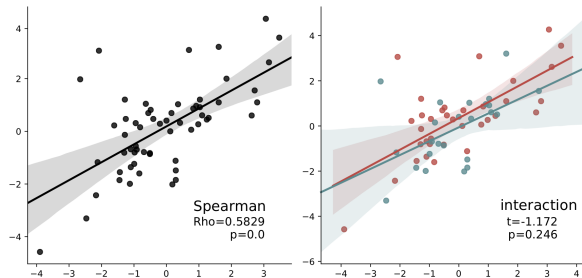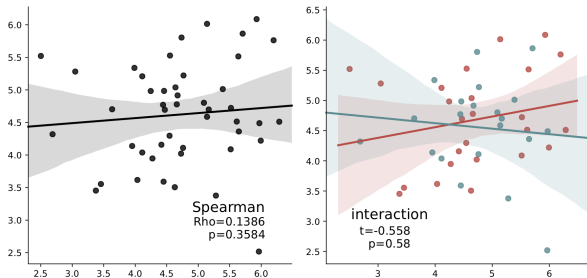

Supplement: FIG S5 [file mSystems.00901-19-sf005.pdf]

A

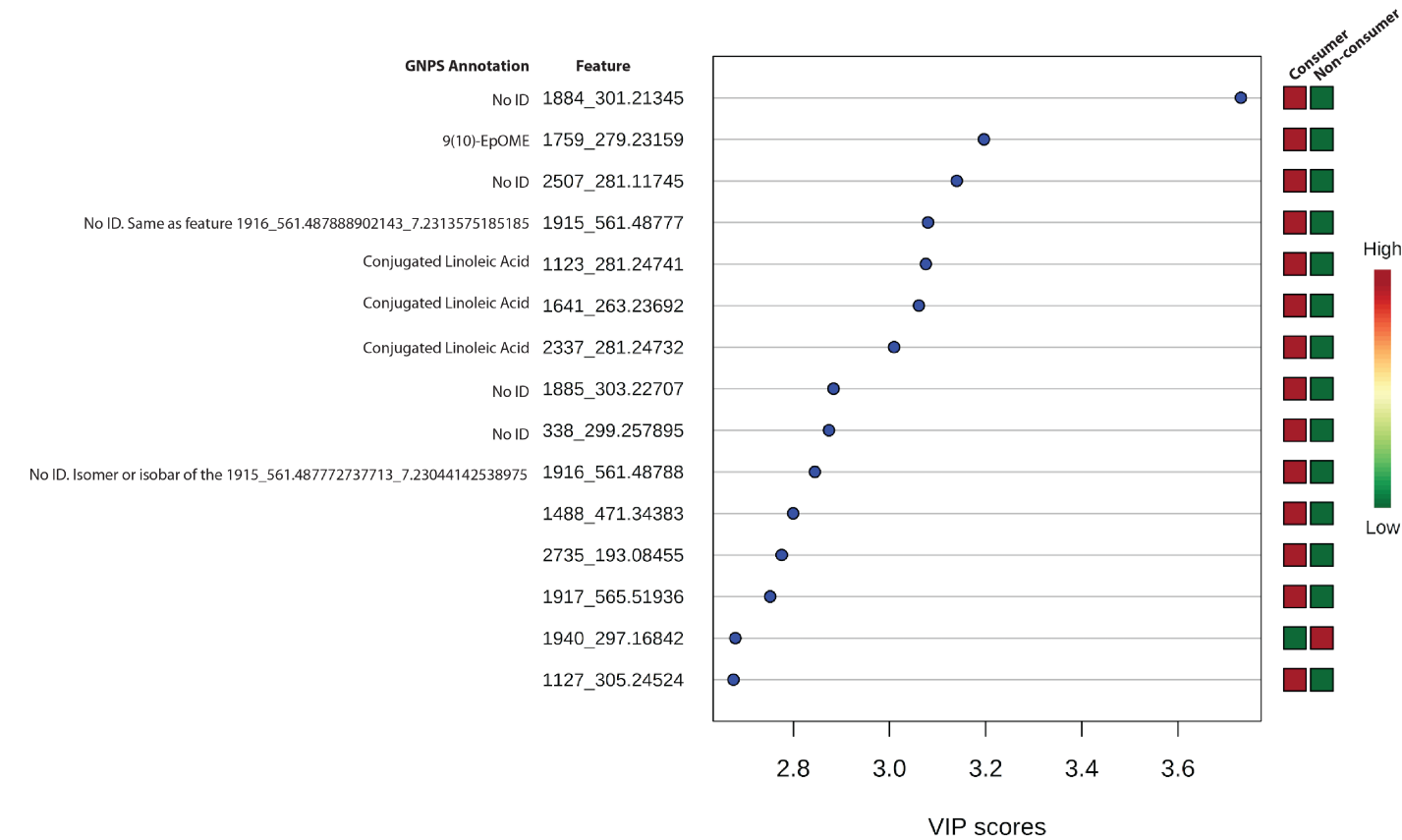

B

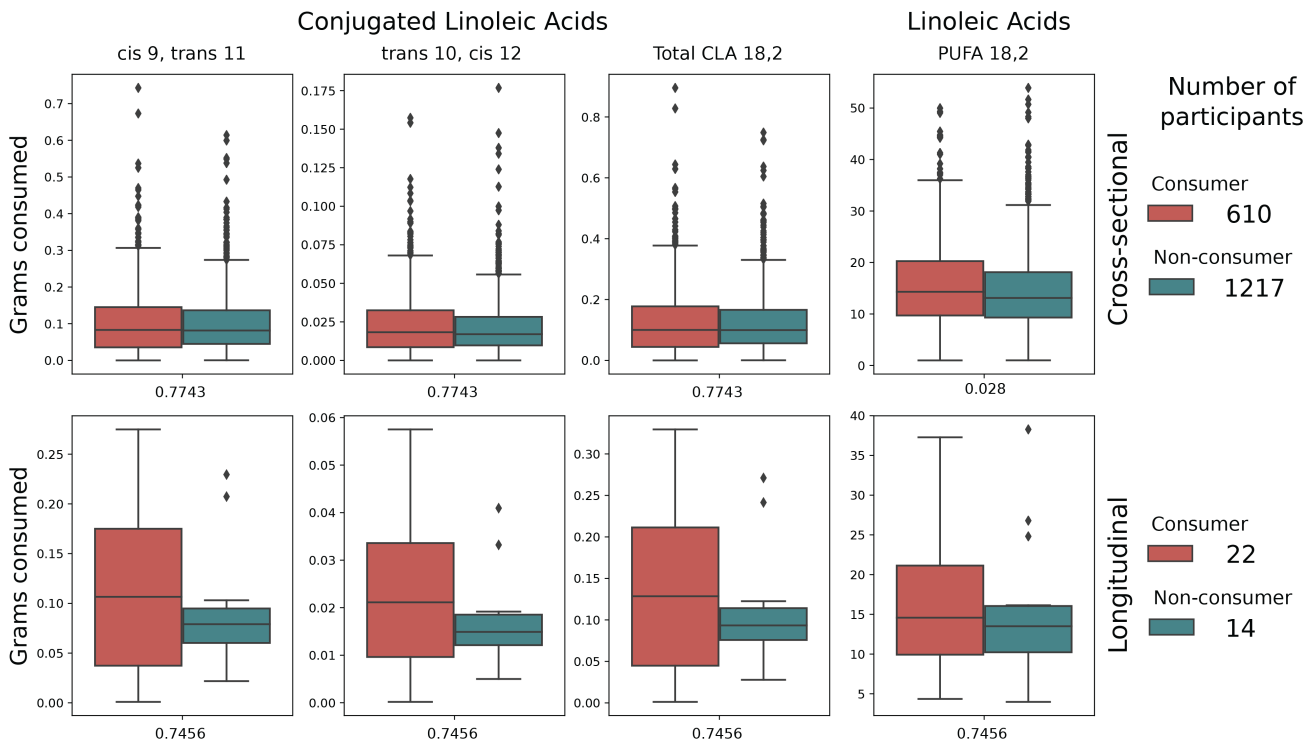

Supplement: FIG S6 [file mSystems.00901-19-sf006.pdf]
