# Supplementary material for: Consumption of Fermented Foods Is Associated with Systematic Differences in the Gut Microbiome and Metabolome
Source: mSystems. 2020 Mar 17;5(2):e00901-19. doi: 10.1128/mSystems.00901-19 (PMC7380580; doi:10.1128/mSystems.00901-19)
Supplement: TABLE S1 [file mSystems.00901-19-st001.pdf]

	Consumer (n = 147)	Non-consumer (n = 163)	p-value
Total energy, kcal/d	1845 ± 615 (605, 3634)	1855 ± 728 (467, 5672)	NS
Total Carbohydrate, *g/d*	173 ± 78 (33, 406)	197 ± 93 (8, 552)	0.012
Total Carbohydrate, % of energy	39 ± 15 (9, 94)	43 ± 13 (1.4, 81)	<0.001
Total Fat, *g/d*	90 ± 44 (5, 239)	80 ± 40 (12, 266)	0.014
Total Fat, % of energy	42 ± 7 (7, 63)	37 ± 5 (23, 48)	<0.001
Total Protein, *g/d*	79 ± 34 (15, 195)	77 ± 45 (18, 457)	NS
Total Protein, % of energy	17 ± 2 (10, 22)	16 ± 2 (15, 32)	<0.001
Total Dietary Fiber, *g/d*	27 ± 12	26 ± 11 (0, 59)	NS
