# Supplementary material for: Consumption of Fermented Foods Is Associated with Systematic Differences in the Gut Microbiome and Metabolome
Source: mSystems. 2020 Mar 17;5(2):e00901-19. doi: 10.1128/mSystems.00901-19 (PMC7380580; doi:10.1128/mSystems.00901-19)
Supplement: TABLE S2 [file mSystems.00901-19-st002.pdf]

Consumer	Non-consumer	Alpha Diversity Metric	H-statistic	p-value
Daily	Rarely	Faith's PD<br>Shannon<br>ObservedOTUs	1.2998<br>1.4434<br>1.328	0.2543<br>0.2296<br>0.2492
Daily	Never	Faith's PD<br>Shannon<br>ObservedOTUs	0.3275<br>0.4501<br>2.1045	0.5671<br>0.5023<br>0.1469
Regularly	Never	Faith's PD<br>Shannon<br>ObservedOTUs	1.4185<br>0.3848<br>0.0451	0.2336<br>0.535<br>0.8317
Occasionally	Never	Faith's PD<br>Shannon<br>ObservedOTUs	0.2723<br>0.0<br>3.1067	0.6018<br>0.9975<br>0.078
