# Supplementary material for: Consumption of Fermented Foods Is Associated with Systematic Differences in the Gut Microbiome and Metabolome
Source: mSystems. 2020 Mar 17;5(2):e00901-19. doi: 10.1128/mSystems.00901-19 (PMC7380580; doi:10.1128/mSystems.00901-19)
Supplement: TABLE S3 [file mSystems.00901-19-st003.pdf]

**Sets**	**Taxa**
Set 1	Lachnospiraceae, *Coprococcus eutacus*, *Cronobacter sakazakii*, *Enterobacteriaceae*, *Pseudomonas stutzeri*, Cyanobacteria order YS2, *Prevotella* spp., *Prevotella copri*, Alphaproteobacteria order RF32, Fusobacteriaceae, *Bacteroides coprophilus*, *Bacteroides* spp., Bacteroidales family S24-7, *Coprococcus* spp.
Set 2	*Bacteroides* spp., *Pseudomonas* spp., *Dorea* spp., Lachnospiraceae, *Prevotella* spp., *Alistipes putredinis*, *Oscillospira* spp., Enterobacteriaceae, *Fusobacterium* spp., *Actinomyces* spp., *Achromobacter* spp., *Clostridium clostridioforme*, *Faecalibacterium prausnitzii*, *Bacteroides uniformis*, Clostridiales, *Delftia* spp.
Set 3	*Lactobacillus acidophilus*, *Lactobacillus brevis*, *Lactobacillus fermentum*, *Lactococcus lactis*, *Leuconostoc mesenteroides*, *Lactobacillus paracasei*, *Lactobacillus plantarum*, and *Lactobacillus rhamnosus.*
Set 4	*Blautia obeum*, *[Eubacterium] hallii*, *Ruminococcus faecis*, *Blautia sp._KLE_1732, Clostridium sp.L2-50, Faecalibacterium prausnitzii, Blautia obeum, Roseburia inulinivorans, Roseburia intestinalis, Bacteroides thetaiotaomicron, [Eubacterium] rectale, Bacteroides ovatus, Bacteroides plebeius, Alistipes putredinis, Bacteroides uniformis, Bacteroides vulgatus, Escherichia coli.*
Set 5	*Streptococcus dysgalactiae, Lachnospiraceae bacterium oral taxon 500, Prevotella melaninogenica, Megamonas hypermegale, Proteus mirabilis, Actinomyces sp. oral taxon 448, Leclercia adecarboxylata, Comamonas kerstersii, Pontibacillus chungwhensis, Citrobacter freundii, Enorma massiliensis, Rhizobium sp. Root651, Enterococcus cecorum, Pseudomonas xanthomarina, Peptoniphilus sp. ChDC B134, Clostridioides difficile, Prevotella multiformis, Lactobacillus kefiranofaciens, Neisseria mucosa, Bifidobacterium psychraerophilum, Lactobacillus brevis, Eubacterium sp. AB3007, Lactobacillus parabuchneri, Bifidobacterium minimum, Prevotella intermedia, Jonquetella anthropi, Lachnospiraceae bacterium M18-1, Lactobacillus acidophilus, Clostridium perfringens, [Clostridium] sporosphaeroides, Streptococcus infantis, Lactobacillus acetotolerans, Bacteroides paurosaccharolyticus, Corynebacterium lactis, Streptococcus mitis, Eggerthella sp. YY7918, Lactobacillus helveticus, Kandleria vitulina, Serratia liquefaciens, Lactobacillus sakei.*
Set 6	*Bacteroides coprophilus, Bacteroides ovatus, Sutterella wadsworthensis, Prevotella bivia, Prevotella copri, Acidaminococcus intestini, Coprobacter fastidiosus, Bacteroides cellulosilyticus, Prevotella stercorea, [Ruminococcus] torques, Dorea longicatena, Sutterella wadsworthensis, Flavonifractor plautii, Finegoldia magna, [Eubacterium] rectale, Roseburia intestinalis, Bacteroides vulgatus, Porphyromonas creviorincanis, Blautia sp. KLE 1732, Escherichia coli, Blautia obeum, Roseburia inulinivorans, [Clostridium] lactatifermentans, Faecalicatena contorta, Alistipes finegoldii, [Clostridium] bolteae, Collinsella intestinalis, Campylobacter hominis, Coprococcus comes, Coprobacter secundus, Eubacterium ventriosum, Bacteroides plebeius, Methanobrevibacter smithii, Parabacteroides distasonis, Tyzzerella nexilis, Bacteroides salanitronis, Clostridium sp. KLE 1755, Collinsella aerofaciens, Kluyvera intermedia, Staphylococcus aureus.*
