# Supplementary material for: Consumption of Fermented Foods Is Associated with Systematic Differences in the Gut Microbiome and Metabolome
Source: mSystems. 2020 Mar 17;5(2):e00901-19. doi: 10.1128/mSystems.00901-19 (PMC7380580; doi:10.1128/mSystems.00901-19)
Supplement: TABLE S4 [file mSystems.00901-19-st004.pdf]

CLA-producer	Feature	mmvecPC1	mmvecRank	#Cons.	#Non-cons.
other	k__Bacteria; p__Bacteroidetes; c__Bacteroidia; o__Bacteroidales; f__Bacteroidaceae; g__Bacteroides; s__Bacteroides_vulgatus	-0.057	6.223	43	36
other	k__Bacteria; p__Bacteroidetes; c__Bacteroidia; o__Bacteroidales; f__Bacteroidaceae; g__Bacteroides; s__Bacteroides_uniformis	-0.031	5.617	43	36
other	k__Bacteria; p__Proteobacteria; c__Gammaproteobacteria; o__Enterobacterales; f__Enterobacteriaceae; g__Escherichia; s__Escherichia_coli	-0.146	5.265	43	36
other	k__Bacteria; p__Bacteroidetes; c__Bacteroidia; o__Bacteroidales; f__Rikenellaceae; g__Alistipes; s__Alistipes_putredinis	-0.181	4.931	43	36
other	k__Bacteria; p__Bacteroidetes; c__Bacteroidia; o__Bacteroidales; f__Prevotellaceae; g__Prevotella; s__Prevotella_copri	-0.099	4.777	24	25
other	k__Bacteria; p__Bacteroidetes; c__Bacteroidia; o__Bacteroidales; f__Bacteroidaceae; g__Bacteroides; s__Bacteroides_ovatus	-0.050	4.643	43	36
other	k__Bacteria; p__Bacteroidetes; c__Bacteroidia; o__Bacteroidales; f__Bacteroidaceae; g__Bacteroides; s__Bacteroides_stercoris	-0.042	4.526	42	36
Eubacterium rectale	k__Bacteria; p__Firmicutes; c__Clostridia; o__Clostridiales; f__Lachnospiraceae; g__; s__[Eubacterium]_rectale	-0.042	4.264	43	36
other	k__Bacteria; p__Bacteroidetes; c__Bacteroidia; o__Bacteroidales; f__Bacteroidaceae; g__Bacteroides; s__Bacteroides_thetaiotaomicron	-0.004	4.244	43	36
other	k__Bacteria; p__Bacteroidetes; c__Bacteroidia; o__Bacteroidales; f__Rikenellaceae; g__Alistipes; s__Alistipes_finegoldii	-0.099	3.744	43	35
other	k__Bacteria; p__Bacteroidetes; c__Bacteroidia; o__Bacteroidales; f__Bacteroidaceae; g__Bacteroides; s__Bacteroides_plebeius	-0.046	3.687	43	36
other	k__Bacteria; p__Firmicutes; c__Clostridia; o__Clostridiales; f__Lachnospiraceae; g__Blautia; s__Blautia_obeum	-0.137	3.640	43	36
other	k__Bacteria; p__Bacteroidetes; c__Bacteroidia; o__Bacteroidales; f__Bacteroidaceae; g__Bacteroides; s__Bacteroides_coprocola	-0.068	3.616	41	36
Roseburia	k__Bacteria; p__Firmicutes; c__Clostridia; o__Clostridiales; f__Lachnospiraceae; g__Roseburia; s__Roseburia_intestinalis	-0.059	3.611	43	36
other	k__Bacteria; p__Bacteroidetes; c__Bacteroidia; o__Bacteroidales; f__Rikenellaceae; g__Alistipes; s__Alistipes_shahii	-0.065	3.579	42	35
other	k__Bacteria; p__Bacteroidetes; c__Bacteroidia; o__Bacteroidales; f__Tannerellaceae; g__Parabacteroides; s__Parabacteroides_distasonis	-0.050	3.573	42	35
Faecalibacterium prausnitzii	k__Bacteria; p__Firmicutes; c__Clostridia; o__Clostridiales; f__Ruminococcaceae; g__Faecalibacterium; s__Faecalibacterium_prausnitzii	-0.062	3.569	43	36
other	k__Bacteria; p__Bacteroidetes; c__Bacteroidia; o__Bacteroidales; f__Bacteroidaceae; g__Bacteroides; s__Bacteroides_cellulosilyticus	-0.027	3.499	43	36

other	k__Bacteria; p__Bacteroidetes; c__Bacteroidia; o__Bacteroidales; f__Barnesiellaceae; g__Barnesiella; s__Barnesiella_intestinihominis	-0.028	3.462	35	32
Roseburia	k__Bacteria; p__Firmicutes; c__Clostridia; o__Clostridiales; f__Lachnospiraceae; g__Roseburia; s__Roseburia_inulinivorans	-0.047	3.419	43	36
Anaerostipes	k__Bacteria; p__Firmicutes; c__Clostridia; o__Clostridiales; f__Lachnospiraceae; g__Anaerostipes; s__Anaerostipes_hadrus	-0.092	3.398	42	35
Roseburia	k__Bacteria; p__Firmicutes; c__Clostridia; o__Clostridiales; f__Lachnospiraceae; g__Roseburia; s__Roseburia_faecis	-0.074	3.389	43	35
other	k__Bacteria; p__Bacteroidetes; c__Bacteroidia; o__Bacteroidales; f__Odoribacteraceae; g__Odoribacter; s__Odoribacter_splanchnicus	0.004	3.329	42	36
other	k__Bacteria; p__Bacteroidetes; c__Bacteroidia; o__Bacteroidales; f__Bacteroidaceae; g__Bacteroides; s__Bacteroides_fragilis	0.009	3.289	42	33
Ruminococcus	k__Bacteria; p__Firmicutes; c__Clostridia; o__Clostridiales; f__Ruminococcaceae; g__Ruminococcus; s__Ruminococcus_bicirculans	-0.047	3.192	37	30
other	k__Bacteria; p__Verrucomicrobia; c__Verrucomicrobiae; o__Verrucomicrobiales; f__Akkermansiaceae; g__Akkermansia; s__Akkermansia_muciniphila	0.045	3.162	26	12
other	k__Bacteria; p__Proteobacteria; c__Betaproteobacteria; o__Burkholderiales; f__Sutterellaceae; g__Sutterella; s__Sutterella_wadsworthensis	-0.116	3.145	17	29
other	k__Bacteria; p__Bacteroidetes; c__Bacteroidia; o__Bacteroidales; f__Tannerellaceae; g__Parabacteroides; s__Parabacteroides_johnsonii	-0.086	2.896	41	36
Eubacterium siraeum	k__Bacteria; p__Firmicutes; c__Clostridia; o__Clostridiales; f__Ruminococcaceae; g__Ruminiclostridium; s__[Eubacterium]_siraeum	0.001	2.881	22	16
other	k__Bacteria; p__Firmicutes; c__Clostridia; o__Clostridiales; f__Oscillospiraceae; g__Oscillibacter; s__Oscillibacter_sp._ER4	0.018	2.857	43	35
other	k__Bacteria; p__Bacteroidetes; c__Bacteroidia; o__Bacteroidales; f__Bacteroidaceae; g__Bacteroides; s__Bacteroides_salyersiae	-0.025	2.841	41	35
Eubacterium	k__Bacteria; p__Firmicutes; c__Clostridia; o__Clostridiales; f__Eubacteriaceae; g__Eubacterium; s__[Eubacterium]_eligens	-0.007	2.816	38	32
other	k__Bacteria; p__Bacteroidetes; c__Bacteroidia; o__Bacteroidales; f__Rikenellaceae; g__Alistipes; s__Alistipes_obesi	-0.043	2.805	39	35
other	k__Bacteria; p__Firmicutes; c__Clostridia; o__Clostridiales; f__Lachnospiraceae; g__Blautia; s__Blautia_sp._KLE_1732	-0.066	2.748	43	36
other	k__Bacteria; p__Firmicutes; c__Clostridia; o__Clostridiales; f__Lachnospiraceae; g__; s__Lachnospiraceae_bacterium_TF01-11	-0.021	2.738	37	34
Roseburia	k__Bacteria; p__Firmicutes; c__Clostridia; o__Clostridiales; f__Lachnospiraceae; g__Roseburia; s__Roseburia_hominis	-0.077	2.589	43	35

other	k__Bacteria; p__Firmicutes; c__Clostridia; o__Clostridiales; f__Oscillospiraceae; g__Oscillibacter; s__Oscillibacter_sp._KLE_1745	-0.042	2.566	42	35
Ruminococcus	k__Bacteria; p__Firmicutes; c__Clostridia; o__Clostridiales; f__Ruminococcaceae; g__Ruminococcus; s__Ruminococcus_faecis	-0.028	2.554	43	36
Eubacterium hallii	k__Bacteria; p__Firmicutes; c__Clostridia; o__Clostridiales; f__Eubacteriaceae; g__Eubacterium; s__[Eubacterium]_hallii	-0.046	2.539	43	36
other	k__Bacteria; p__Firmicutes; c__Clostridia; o__Clostridiales; f__Lachnospiraceae; g__Coprococcus; s__Coprococcus_comes	-0.051	2.419	43	35
other	k__Bacteria; p__Bacteroidetes; c__Bacteroidia; o__Bacteroidales; f__Prevotellaceae; g__Prevotella; s__Prevotella_sp._109	0.001	2.404	41	35
other	k__Bacteria; p__Firmicutes; c__Clostridia; o__Clostridiales; f__Lachnospiraceae; g__Dorea; s__Dorea_longicatena	-0.038	2.403	41	35
other	k__Bacteria; p__Firmicutes; c__Clostridia; o__Clostridiales; f__Lachnospiraceae; g__Tyzzerella; s__Tyzzerella_nexilis	-0.058	2.388	43	35
Bifidobacterium adolescentis	k__Bacteria; p__Actinobacteria; c__Actinobacteria; o__Bifidobacteriales; f__Bifidobacteriaceae; g__Bifidobacterium; s__Bifidobacterium_adolescentis	-0.035	2.342	30	25
other	k__Bacteria; p__; c__; o__; f__; g__; s__bacterium_LF-3	-0.045	2.281	39	32
other	k__Bacteria; p__Bacteroidetes; c__Bacteroidia; o__Bacteroidales; f__Bacteroidaceae; g__Bacteroides; s__Bacteroides_coprophilus	-0.011	2.169	42	35
other	k__Bacteria; p__Proteobacteria; c__Betaproteobacteria; o__Burkholderiales; f__Sutterellaceae; g__Parasutterella; s__Parasutterella_excrementihominis	-0.029	2.130	33	28
other	k__Bacteria; p__Firmicutes; c__Negativicutes; o__Veillonellales; f__Veillonellaceae; g__Dialister; s__Dialister_invisus	-0.034	2.114	14	12
Clostridium	k__Bacteria; p__Firmicutes; c__Clostridia; o__Clostridiales; f__Clostridiaceae; g__Clostridium; s__Clostridium_sp._L2-50	-0.108	2.113	43	36
Eubacterium	k__Bacteria; p__Firmicutes; c__Clostridia; o__Clostridiales; f__Eubacteriaceae; g__Eubacterium; s__Eubacterium_ramulus	-0.041	1.975	43	34
